# Flowers and Beauty

**Published:** 1871-11

**Authors:** 


### FLOWERS AND BEAUTY.

During the war, when so many of our noble soldiers were pining away in dreary hospitals, we recommended that some pretty girls with baskets of flowers should pass through the hospitals at a certain hour every day. Wonder if from that hint the ten thousand bouquets of flowers were sent to the hospitals this season by the kind hearted ladies of New York city? If so, we have not lived in vain.

But youth and beauty or matronly elegance should have accompanied every flower, for the reason that soldiers are brimful of gallantry and human nature too. Flowers remind us of the innocence of boyhood's home; a pretty girl, of the angels; and a lady, of mother, and wife, and sister at home; and girl, and flowers, and matron, are by their very sight, elevating, purifying, healing. It is said that during the Crimean War, the daily visits of Florence Nightingale were looked forward to from the very hour in which she passed; that the soldiers strove to put themselves within her shadow, when they could do nothing better, and that the keepers observed a general tidying up of things as the hour of her visit approached. Then again there is a healing in the gentle touch and the soft tone of inquiry and sympathy of a lady; there are cases in which giver and receiver are both blessed; the one made no poorer, the other made richer, and comforted, O, how much!

Let flowers be placed before the bar of the prisoner, in the window of the lunatic, and arrange that the unfortunate dwellers in the penitentiary shall look upon a flower bed from their dismal sleeping places. And as to the last named, it would be a wise enactment that tasks should be appointed in all cases, and that they should be paid in money for all over-work, to be saved for them or their families; so that when their term expired they would have something to begin the world with, and not have to resort to criminal acts on the first day of their deliverance, to keep them from starving. This fund ought to be increased by the offer of pecuniary rewards for good conduct and meritorious deeds, and, in addition, the term of imprisonment to be reduced in proportion to fidelity. Reader, the prisoner is our brother still.

